# Migratory Patterns of Wild Chinook Salmon *Oncorhynchus tshawytscha* Returning to a Large, Free-Flowing River Basin

**DOI:** 10.1371/journal.pone.0123127

**Published:** 2015-04-28

**Authors:** John H. Eiler, Allison N. Evans, Carl B. Schreck

**Affiliations:** 1 Auke Bay Laboratories, Alaska Fisheries Science Center, National Marine Fisheries, National Oceanic and Atmospheric Administration, Juneau, Alaska, United States of America; 2 Department of Fisheries and Wildlife, Oregon State University, Corvallis, Oregon, United States of America; 3 U.S. Geological Survey, Oregon Cooperative Fish and Wildlife Research Unit, Department of Fisheries and Wildlife, Oregon State University, Corvallis, Oregon, United States of America; University of California, Berkeley, UNITED STATES

## Abstract

Upriver movements were determined for Chinook salmon *Oncorhynchus tshawytscha* returning to the Yukon River, a large, virtually pristine river basin. These returns have declined dramatically since the late 1990s, and information is needed to better manage the run and facilitate conservation efforts. A total of 2,860 fish were radio tagged during 2002–2004. Most (97.5%) of the fish tracked upriver to spawning areas displayed continual upriver movements and strong fidelity to the terminal tributaries entered. Movement rates were substantially slower for fish spawning in lower river tributaries (28–40 km d^-1^) compared to upper basin stocks (52–62 km d^-1^). Three distinct migratory patterns were observed, including a gradual decline, pronounced decline, and substantial increase in movement rate as the fish moved upriver. Stocks destined for the same region exhibited similar migratory patterns. Individual fish within a stock showed substantial variation, but tended to reflect the regional pattern. Differences between consistently faster and slower fish explained 74% of the within-stock variation, whereas relative shifts in sequential movement rates between “hares” (faster fish becoming slower) and “tortoises” (slow but steady fish) explained 22% of the variation. Pulses of fish moving upriver were not cohesive. Fish tagged over a 4-day period took 16 days to pass a site 872 km upriver. Movement rates were substantially faster and the percentage of atypical movements considerably less than reported in more southerly drainages, but may reflect the pristine conditions within the Yukon River, wild origins of the fish, and discrete run timing of the returns. Movement data can provide numerous insights into the status and management of salmon returns, particularly in large river drainages with widely scattered fisheries where management actions in the lower river potentially impact harvests and escapement farther upstream. However, the substantial variation exhibited among individual fish within a stock can complicate these efforts.

## Introduction

Migratory behavior is exhibited by numerous animal species and is recognized as an important biological parameter [1], [2]. The significance of this behavior is further demonstrated by its prominence across a wide range of taxa and life history strategies in spite of the associated energetic costs, which can be substantial particularly for animals exhibiting extended movements [3]. Ironically, the migratory patterns exhibited are often not well understood. This is particularly true for fish [4] due in part to the difficulties associated with collecting information in the aquatic environment where access and visual observations are limited. Movement data are also complex, since they encompass both spatial and temporal aspects of an animal’s life history. Unlike the well-established methods used to estimate abundance, survival, and mortality, efforts to describe and quantify animal movements have often lagged noticeably behind [5].

Fish migrations are generally defined as cyclical and directed movements by large segments of a population actively swimming extended distances between separate and distinct habitats [6]. These migratory movements typically occur within a predictable period of time, and serve to bring populations into contact with resources that either enhance or are essential for growth, survival, or reproduction. Pacific salmon *Oncorhynchus* spp. migrations have received considerable attention due to the large numbers of fish involved and cyclical nature of the returns, the extended distances traveled, and the economic and biological importance of this resource.

The anadromous life history and associated seaward migration exhibited by salmon provide an opportunity for the juvenile fish to escape from the relatively sterile habitats of their natal streams in favor of more productive conditions in the marine environment where food is more readily available [7]. However, this strategy carries with it the burden of having to return to freshwater to spawn. The extreme physical demands associated with this journey present the fish with a migratory dilemma. In addition to being in good physical condition, the fish must exhibit the migratory timing and swimming behaviors that enable them to return to suitable rivers, migrate upstream, and arrive on spawning grounds when conditions are favorable. At the same time, the fish must also conserve energy to ensure that they reach their final destination with sufficient reserves to reproduce. To be successful, the migratory patterns of the fish must achieve a balance between these two competing requirements.

In addition to these demands, both environmental and anthropogenic factors (*e*.*g*. man-made structures that impede migratory movements, industrialization and other activities that alter existing habitats) are increasingly impacting salmon populations in rivers throughout their range [8], [9], underscoring the need to better understand the underlying characteristics of these returns. Although recognized as an important life history parameter, detailed information on fish movements in large rivers is often limited due to the effort and costs associated with implementing large-scale monitoring programs over extended distances and periods of time. Advances in biotelemetry, including the development of equipment systems robust enough to track large numbers of highly mobile individuals over vast areas, have substantially enhanced the ability to collect movement data and have been used effectively on migrating salmon [10], [11], [12], [13]. This approach has the capacity to substantially increase our understanding of salmon movements on a scale large enough to be useful to fishery managers and behavioral ecologist interested in long-distance migrations.

Sizeable numbers of Chinook salmon *O*. *tshawytscha* return to the Yukon River, a large northern river basin in Alaska and northwestern Canada. Abundance estimates during 2002–2004 ranged from approximately 125,000 to 262,000 fish annually [14]. These returns are composed of multiple stocks distributed throughout the basin [15], support important commercial and subsistence fisheries in both the United States and Canada [16], and are an integral part of the Yukon River ecosystem. The upriver movements of the fish are characterized by long-distance migrations. Whereas fish returning to lower river tributaries may only travel several hundred kilometers upriver from saltwater and arrive on spawning grounds within several weeks, some upper basin stocks travel over 3,200 km and take over 60 days to reach their final destination. Fish returning to the upper headwaters exhibit some of the longest freshwater migrations on record.

Chinook salmon returns to the basin were reasonably stable until the late 1990s when dramatic declines in abundance were reported [17], [18]. This trend has continued during subsequent years, and resulted in the closure or drastic reductions in commercial fishing, severe restrictions in subsistence harvests, and difficulties in meeting regional and basin-wide escapement goals [16], [19]. In response to these events, a basin-wide telemetry study was conducted during 2002–2004 to determine the stock composition, timing, and spawning distribution of the returns [15]. The study also provided detailed information on the upriver movements of the fish. Understanding the migratory patterns—in combination with information on stock structure and run timing—can provide numerous insights into the status and management of salmon returns. Movement data can be particularly important in large river drainages with widely scattered fisheries where management actions in the lower river potentially impact harvests and escapement farther upstream.

In this paper, we compare the regional, stock-specific, and individual movement rates and migratory patterns of Chinook salmon returning to spawning areas throughout the Yukon River basin. In a companion paper with analyses modeled after [20], we used the migratory patterns described in this paper to evaluate the factors that potentially affect the upriver movements of the fish, including the physical features of the basin and the biological characteristics of the fish. The movements of large aggregates of fish and less common migratory patterns are also examined in the current paper.

The information we present is unique in several ways, representing one of the few comprehensive, basin-wide movement studies undertaken on such a vast spatial scale. The Yukon River is one of the largest drainages in North America, both in terms of size and discharge [21], [22]. Despite the logistical challenges, sizeable numbers of fish were radio tagged and tracked upriver, making meaningful comparisons possible for fish traveling varying distances. Many large rivers with sizable Chinook salmon returns are heavily regulated with controlled flows and impounded reaches, and the returns frequently comprised of both wild and hatchery fish. In contrast, the Yukon River is virtually pristine and essentially free-flowing, and the returns composed almost exclusively of wild stocks, providing an opportunity to document the migratory patterns of the fish under natural conditions and across heterogeneous environments. The results of this study provide a useful comparison with information from altered or impaired river systems, as well as other unaffected river drainages where movement data is not readily available.

## Materials and Methods

### Ethics Statement

The fish handled during this study were not anesthetized, euthanized, or sacrificed. The handling methods used were reviewed by the Joint Technical Committee of the U.S.-Canada Yukon River Panel, and conform to National Marine Fisheries Service standards. The study was authorized by the State of Alaska under cooperative research plans for 2002–2004.

### Study Area

**Fig 1:**
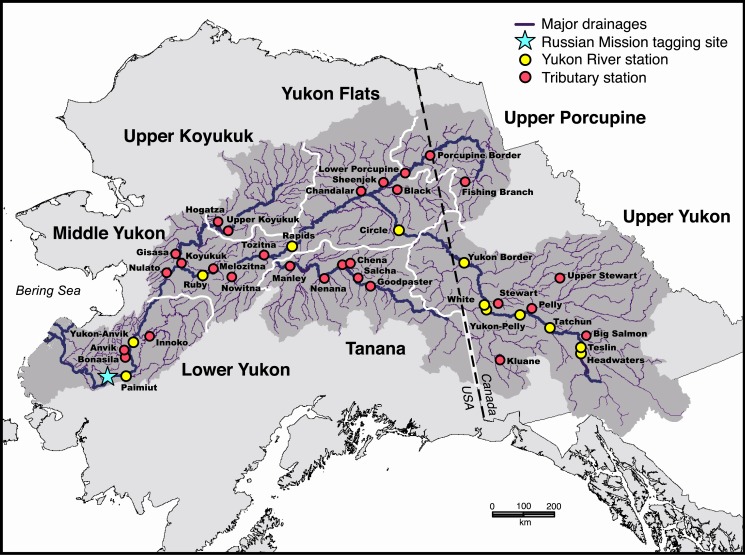
Map of the Yukon River basin showing the regional areas, major drainages, lower river tagging site near Russian Mission, and tracking stations on both the Yukon River main stem and associated tributaries.

The Yukon River basin drains a watershed of more than 855,000 km^2^. The main river alone flows for more than 3,000 km from its headwaters in Canada to the Bering Sea (**Fig 1**). The river is deep, with channel depths exceeding 20 m in the lower basin compared to 12–14 m downstream of the Yukon-Tanana River confluence and 5–7 m near the U.S.-Canada border (distances of ~ 1,100 and 2,000 km from the river mouth, respectively). In addition to its immense size, the Yukon River is the fifth largest drainage in North America in terms of total annual discharge, and exhibits considerable temporal variability with greater discharge during the summer months[21], [22]. The river is essentially free-flowing. Only a small, passable hydroelectric dam located ~ 2,800 km upriver from the river mouth impedes the natural flow of water. Less than 3% of the Chinook salmon return travel past this site, and a naturally-occurring lake 35 km downstream minimizes any impact that might be caused by the restricted flow on fish movements.

Several major tributaries flow into the Yukon River main stem (hereafter referred to as the main stem). The basin also includes numerous medium and small-sized tributaries (**Fig 1**). Most reaches of the basin consist of a primary river channel with occasional side channels and sloughs, although the main stem is extensively braided in the area commonly referred to as the Yukon Flats (**Fig 1**). Sections of the Tanana River, White River, and Canadian main stem are also noticeably braided. Water visibility in many areas is extremely poor, particularly in the Tanana and White rivers due to turbidity from glacial activity in the upper headwaters of these drainages.

The basin was subdivided into seven regions, including the Lower Yukon, Middle Yukon, Upper Koyukuk, Tanana, Yukon Flats, Upper Porcupine, and Upper Yukon (**Fig 1**). Regional designations were based on geographic location and the general geomorphology of the area; for example, lower reaches of the Porcupine River were considered part of the Yukon Flats due to proximity and similarities in landscape and river characteristics. The Yukon River basin is extremely remote, with access to most areas limited to boat or aircraft.

Although harvests have been severely restricted in recent years, Chinook salmon are a major source of food in many remote communities and provide a source of income for local residents. Subsistence and commercial fisheries occur throughout the basin with most fishing effort concentrated near villages along the main stem [16]. Fish are also harvested in a number of tributaries. Limited sport fishing takes place within the basin.

### Tagging and Tracking

Details regarding the methods used to capture and tag the fish have been previously described [15]. Briefly, adult Chinook salmon were captured from early June to mid-July with drift gill nets in the lower Yukon River near the village of Russian Mission located 303 km upriver from the Yukon River mouth (**Fig 1**). During 2002, fish were also captured near the village of Marshall, located approximately 90 km downstream from the principal tagging area. Both day (0900–1700 hours) and night (1800–0200 hours) shifts were fished during the study.

The fish were tagged with pulse-coded radio transmitters in the 150–151 MHz frequency range (manufactured by Advanced Telemetry Systems, Isanti, MN), which were gently inserted through the mouth and into the stomach. The physical dimensions and signal characteristics of the transmitters are previously described [15]. The transmitters were also equipped with a motion sensor and activity monitor similar to those described by Eiler [23] and used to determine the real-time status of the fish (i.e., actively moving, active during the previous 24 hours, or inactive during this period). The fish were marked externally with spaghetti tags attached just below the dorsal fin as described by Wydoski and Emory [24] to help identify tagged individuals caught in fisheries or located in spawning areas.

Radio-tagged fish that moved upriver were tracked with remote tracking stations [25], [26] placed at 40 sites throughout the basin (**Fig 1**). The sites were located on important migratory routes and major tributaries. Pairs of stations were placed at sites with special significance, including Paimiut, lower Koyukuk River, Manley, Rapids, Yukon Border, and Porcupine Border, to avoid loss of data due to technical problems with the equipment, damage from bears *Ursus* spp., or other unforeseen difficulties. The stations consisted of several integrated components, including a data-logging receiver (Advanced Telemetry Systems), satellite uplink (Campbell Scientific, Logan, UT), directional receiving antennas oriented upriver and downriver to provide information on the general location of the fish in relation to the site, and a self-contained power system [26].

Fish within reception range were identified and recorded by the stations. The information collected included the date and time the fish were present at the site, signal strength of the transmitter, and the orientation of the fish in relation to the station (*i*.*e*. upriver or downriver from the site). The information was recorded and summarized at 10-minute intervals. The methods used to transmit, access, and evaluate the station data are previously described [15]. Periodic aerial surveys were conducted to locate fish between station sites and upriver of stations on terminal tributaries based on the real-time information provided by the stations (e.g., the last known location of the fish). Fish were tracked from fixed-wing aircraft and helicopters as described by Eiler [26]. Helicopters were also used to access remote areas to determine the status of the fish and recover transmitters. Tracking receivers equipped with an integrated global positioning system (GPS) receiver were used during the surveys to standardize the location records of the fish.

### Assessing Upriver Movements

Fish that passed Paimiut (the first station site, located approximately 62 km upriver from Russian Mission) were considered to have resumed their upriver migration after being tagged. Fish tracked to terminal tributaries were designated as members of the spawning stock associated with that tributary (shown in **S1 Fig**). Genetic stock identification (GSI) estimates, derived from tissue samples taken from the tagged fish, were used to validate this assumption [27]. Fish last located in non-terminal areas or harvested in mainstem fisheries were excluded from the analysis since they were potentially destined for spawning areas farther upriver, although the movements of these fish were summarized for comparative purposes (**S2 Fig**). Local fishers were asked to report any radio-tagged fish they caught. Fish located out of water (based on signal strength and reception range) in villages or fish camps during aerial surveys were considered to have been harvested even if the recovery was not reported.

The tracking records of each fish were systematically reviewed post-season to verify passage by the station sites and to confirm that the movements corresponded to a sequential series of stations. The time of passage (*i*.*e*. date and time the fish moved past the site) was determined by comparing the progressive change in signal strength of the transmitter detected by the station’s directional antennas and was designated as the time that the strongest signal shifted from the downriver antenna to the upriver antenna. Fish that continuously moved upriver and remained in the terminal tributary they entered were deemed to be exhibiting typical migratory movements. Fish that deviated from this pattern (hereafter referred to as atypical movements, which denotes migratory patterns that were less common rather than abnormal or affected behavior) were considered separately. The frequency of atypical movements was compared by region and terminal tributary (stock).

#### Movement rates

Average movement rates (km d^-1^) were determined for the fish that traveled past Paimiut and had complete tracking records (*i*.*e*. recorded by all the stations along their migratory route). The rates were based on the distance traveled (between Paimiut and the last station) divided by the time taken to travel between the two sites. Movements between the tagging site and Paimiut were excluded from the analysis to avoid incorporating tagging-induced behavior that could bias the results [15]. Travel time was calculated using the date and time of passage at each site. Distance was estimated using an ArcGIS mapping program [28]. Due to the size of the basin and scope of the study, it was not possible to determine the actual pathway selected by the fish. Therefore, distance was based on the assumption that fish were primarily traveling along the thalweg. Average movement rate by region and stock was calculated as the mean of the average movement rates of the individual fish returning to these areas.

A similar approach was used to calculate movement rates between sequential stations. These rates were based on the distance traveled between the two stations and the time taken to reach the second site. Average movement rates between sequential stations were also calculated by region and stock.

#### Migratory patterns

Migratory patterns were defined as the series of sequential movement rates exhibited by the fish in successive reaches of the basin. Movement rates between the Paimiut and Yukon-Anvik River stations (**Fig 1**) were used to represent the initial phase of the migratory pattern, with the remainder described by movement rates in subsequent reaches. Regional and stock-specific migratory patterns were determined for fish returning to each area. There were few mainstem stations in the Lower Yukon; most of the stations in this region were located on terminal tributaries. Consequently, the information on Lower Yukon fish is limited to stock-specific comparisons.

The migratory patterns of individual fish within a stock were compared using nonmetric multidimensional scaling (NMS), a nonparametric, multivariate ordination technique [29], [30] used to identify the migratory characteristics that best explained the individual variation exhibited by the fish. Based on the movement rates observed in sequential reaches, fish were ranked along several gradients (representing different sources of variation within the data) to identify individuals with similar and disparate migratory patterns. The NMS approach was selected over other ordination methods because it can accommodate non-normal or discontinuous data, does not assume linearity, is based on ranked distances, which improves its ability to extract information from nonlinear relationships, can be used with any distance measure or data transformation, and is generally considered the most effective ordination method for ecological data [31]. Separate ordinations were conducted for each stock (*i*.*e*. within-stock analyses). Stocks with a minimum of three stations along their migratory route and represented by ≥ 20 radio-tagged fish were analyzed. Only fish recorded by all stations along the migratory route and ultimately located at spawning sites within the terminal tributary were included in the sample. The main data matrix used in the analysis consisted of individual fish (rows) by stations along the migration route of the stock (columns), with the cells denoting the fish’s movement rate for the reach.

The original multivariate data (*i*.*e*. sequential movement rates of the individual fish) were reduced to a small number of continuous synthetic variables (axes) representing the variation gradients that best characterized the differences exhibited by the individual fish. The NMS method iteratively searched for an ordination with low stress as measured by the relationship between ranked distances in the original multidimensional space and those in the reduced space containing fewer dimensions [32]. The ordination was conducted with PC-ORD software (MjM Software Design, Gleneden Beach, OR) using the “slow and thorough” autopilot mode to determine the minimum stress value from 250 runs for solutions containing up to six-dimensions. A Monte Carlo test was performed using 250 runs of randomized data to determine if the ordination solution provided significantly more reduction in stress than expected by chance (α ≈ 0.05). Euclidean distance measurements [31] were used to calculate the dissimilarity matrix. This metric was selected because movement data is continuous (*i*.*e*. all matrix combinations were possible), and absolute differences (versus proportional differences) in movement rate, which were our primary interest, are adequately reflected by this measure.

The synthetic axes created by NMS were interpreted using Pearson’s correlation (*r*), Kendall’s nonparametric rank correlation (τ), and scatter plots of the data to characterize the relationship between movement rates at sequential stations and the axis scores of the individual fish. The percentage of the variation in the original data represented by the ordination was calculated with Pearson’s coefficient of determination (*r* ^*2*^).

#### Fish pulse progression

The number of Chinook salmon moving through the lower river during the return exhibited a series of distinct and sizable peaks, hereafter referred to as fish pulses. Based on catch per unit effort (CPUE) data from the Russian Mission tagging site, the pulses were observed at varying times over the course of the run and were discrete, with most only lasting several days [15]. Tagging efforts were intensified during a pronounced pulse in 2003 to determine whether this group of fish exhibited synchronous movements upriver. The number of days for the tagged fish to collectively move past sites farther upriver was determined by comparing the passage dates of the first and last tagged fish recorded at successive stations along the migratory route.

## Results

### Tagging and Tracking

Fishing commenced in early June and continued until the end of the run in mid-July when catch rates were low. Large numbers of Chinook salmon (N = 2,860) were captured and radio tagged, with transmitters deployed throughout the run. The final destinations of the 2,790 fish tracked past Paimiut have been previously reported [15]. Of the 2,626 fish tracked past multiple stations, 2,560 fish (97.5%) displayed typical upriver movements (**Table 1**). Atypical movements were exhibited by the 66 remaining fish (2.5%) and are described separately.

**Table 1:** Tagging dates and numbers of Chinook salmon capture in the lower Yukon River, radio tagged, and tracked upriver passed the first tracking station site (Paimiut) during 2002–2004.

Category	2002	2003	2004	All years
Start of tagging	9 June	3 June	3 June	3–9 June
End of tagging	13 July	14 July	19 July	13–19 July
Captured	1,310	2,312	2,107	5,729
Tagged	768	1,097	995	2,860
Moved upriver past Paimiut	751	1,081	958	2,790
Tracked past multiple stations	683	1,050	893	2,626
Typical migratory pattern	666 (97.5)	1,031 (98.2)	863 (96.6)	2,560 (97.5)
Regional fish^1^	242	470	385	1,097
Terminal tributary fish^2^	196	394	287	877
Atypical migratory pattern	17 (2.5)	19 (1.8)	30 (3.4)	66 (2.5)

The numbers of fish exhibiting typical (only upriver movements) and atypical migratory patterns are presented. The percentage of the fish that moved upriver and were recorded by multiple stations is in parentheses.

^1^Fish exhibiting the typical migratory pattern, tracked to terminal tributaries or harvested in main river fisheries in terminal regions, and recorded by all stations along the migratory route.

^2^Fish exhibiting the typical migratory pattern, tracked to terminal tributaries, and recorded by all stations along the migratory route.

Fish length averaged 833 mm (ranging from 395 to 1,075 mm) and was similar across years based on box plots of the data. The fish length was generally similar across stocks, with most having median lengths between 800 and 900 mm. Other physical characteristics of the fish are reported separately [15].

### Movement Rates

Movement rates were determined for the regional components of the return based on 1,097 fish with complete tracking records. A total of 877 fish were tracked to terminal tributaries and used to describe the upriver movements of specific stocks. The average movement rate of these fish was 51 km d^-1^ (SE = 0.37), but pronounced differences were observed among regions, stocks, individual fish, and specific reaches of the basin. Substantially slower rates were observed for fish returning to terminal tributaries lower in the basin (**Table 2**). Average movement rates for Lower Yukon stocks ranged from 28–40 km d^-1^ compared to 45–46 km d^-1^ for Tanana stocks, and 52–62 km d^-1^ for fish returning to the Yukon Flats, Upper Porcupine, and Upper Yukon, hereafter referred to collectively as the upper basin. Middle Yukon fish exhibited movement rates that were generally faster than Lower Yukon fish, but considerably slower than upper basin stocks. Upper Koyukuk fish displayed the fastest movement rates, averaging 66 km d^-1^. Although the Yukon-Koyukuk River confluence is low in the basin compared to other major tributaries (420 km upriver from Paimiut), these fish traveled to spawning areas in the upper reaches of the drainage, distances in excess of 1,200 km upriver from Paimiut.

**Table 2:** Average movement rate (km d^-1^) of radio-tagged Chinook salmon with complete tracking records (recorded by all tracking stations along their migratory route) returning to terminal tributaries in the Yukon River basin during 2002–2004.

Region	Stock	Stations	N	Rate	SE	CV (%)
Lower Yukon	Bonasila	2	22	39.5	2.6	31.1
	Anvik	2	50	27.9	1.6	39.6
Middle Yukon	Nulato	3	27	39.1	2.1	28.2
	Gisasa	4	13	41.3	2.3	19.8
	Tozitna	4	16	50.5	2.7	21.3
Upper Koyukuk	Koyukuk^1^	4	23	65.7	1.3	9.3
Tanana	Kantishna	4	21	54.8	1.1	9.4
	Chena	6	56	45.8	1.0	15.5
	Salcha	6	96	45.2	0.5	11.4
	Goodpaster	6	52	46.0	0.7	10.9
Yukon Flats	Chandalar	5	26	56.7	1.5	13.8
	Sheenjek	5	10	56.3	0.9	5.3
Upper Porcupine	Canadian stocks^2^	6	30	59.6	1.6	15.1
Upper Yukon	Klondike	6	24	61.6	0.9	7.2
	Stewart	7	56	57.2	0.9	11.1
	White	6	24	59.2	0.7	5.6
	Pelly	9	97	57.4	0.5	9.0
	Little Salmon	9	11	52.6	2.0	12.9
	Big Salmon	10	56	53.8	0.6	7.9
	Teslin	10	81	54.5	0.7	10.8
	Headwaters^3^	10	19	52.2	0.8	7.0

Stocks represented by less than 10 fish are not listed. The number of tracking stations along the migratory route, number of fish, average movement rate, standard error (SE) and coefficient of variation (CV) are indicated for the principal stocks of the return.

^1^ Composite of headwater stocks, including Henshaw, South Fork, and Middle Fork rivers.

^2^ Primarily Miner River fish, but also including fish returning to the Old Crow River and Whitestone River.

^3^ Including fish returning to the Takhini River and other headwater tributaries.

Average movement rates were more variable for stocks returning to the lower reaches of the basin. The coefficient of variation for Lower Yukon and Middle Yukon stocks ranged from 31–40% and 20–28%, respectively. Less variation was observed for Upper Koyukuk (9%), Tanana (9–16%), and upper basin (5–15%) stocks (**Table 2**).

### Migratory Patterns

#### Regional differences

**Fig 2:**
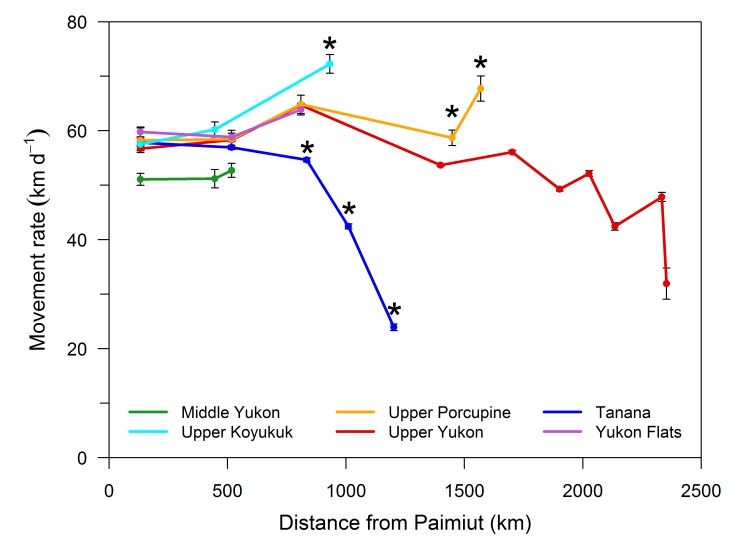
Movement rates for Chinook salmon returning to regional areas of the Yukon River basin during 2002–2004. Estimates and standard error are based on fish with complete tracking records (recorded by all stations passed). Movement rates for fish that have left the Yukon River main stem are also indicated (*). Regional estimates were not available for Lower Yukon stocks due to the limited number of Yukon River stations in this region.

**Fig 3:**
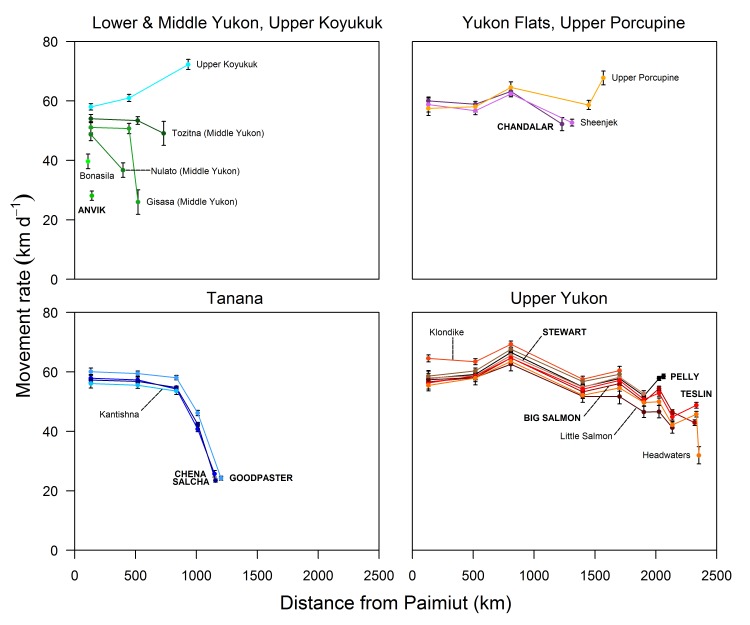
Movement rates for the principal Chinook salmon stocks returning to regional areas of the Yukon River basin during 2002–2004. Estimates and standard error are based on fish with complete tracking records (recorded by all stations passed). Capitalized stock names indicate major stocks within the basin.

The migratory patterns of the fish showed distinct regional differences in relation to distance traveled and the nature of the river (*e*.*g*. main stem vs. tributary reach). Regional aggregates of fish traveling shorter distances to reach their terminal tributaries (Lower Yukon and Middle Yukon) were uniformly slower than those traveling farther upriver (**Figs 2 and 3**). Conversely, movement rates in the lower reaches of the main stem (< 550 km upriver from Paimiut) were remarkably similar for stocks returning to the Upper Koyukuk, Tanana, and upper basin (57–60 km d^-1^) despite the disparate distances ultimately traveled by these fish to reach their terminal tributaries. However, conspicuous differences were observed as the fish moved farther upriver. Three distinct migratory patterns were identified, including a 1) substantial increase in movement rate after leaving the main stem, 2) pronounced and consistent decline in movement rate after leaving the main stem, and 3) gradual but erratic decline in movement rate associated with extended mainstem migrations.

The first migratory pattern was exhibited by fish returning to the Upper Koyukuk and Upper Porcupine. Upper Koyukuk fish swam considerably faster (averaging 72 km d^-1^) after leaving the main stem (**Fig 2**). Upper Porcupine fish displayed a similar pattern, with comparable movement rates while traveling through mainstem reaches relative to the other upper basin stocks, followed by a pronounced increase in swimming speed (68 km d^-1^) after leaving the main stem. Ancillary information (based on estimated movement rates for several fish recovered in fisheries and assessment projects in the upper reaches of the drainage) suggests that Upper Koyukuk fish may exhibit a reduction in movement rate as they near their terminal tributaries. Comparable information was not available for fish returning to the Upper Porcupine.

The second migratory pattern was exhibited by the Tanana component of the return. Although movement rates downriver of the Yukon-Tanana River confluence were comparable to upper basin stocks, Tanana fish displayed a pronounced and consistent decline in movement rate after leaving the main stem, with movement rates declining from 57 to 24 km d^-1^ as the fish approached at their terminal tributaries (**Fig 2**). A similar migratory pattern was observed for Middle Yukon fish returning to the Gisasa River, located in the lower reaches of the Koyukuk River. Movement rates for these fish declined from 51 to 26 km d^-1^ after leaving the main stem (**Fig 3**).

The third migratory pattern (gradual but erratic decline in movement rate) was exhibited by fish returning to the Yukon Flats and Upper Yukon. Although movement rates initially increased after the fish passed the Yukon-Tanana River confluence (743 km upriver from Paimiut), decreases in swimming speed were subsequently observed as the fish traveled farther upriver. Movement rates periodically increased following these declines, but the overall trend was toward slower swimming speeds (**Figs 2 and 3**). Despite this trend, the movement rates of Yukon Flats and Upper Yukon fish were still fast even when nearing their final destination (**Fig 3**). Stocks returning to the Yukon Flats averaged from 52 to 53 km d^-1^ as they approached their terminal tributaries. These stocks traveled less than 230 km upriver after passing their final station to reach spawning areas, distances comparable to those exhibited by the principal Tanana stocks (**Table 3**). Similar movement rates were observed for Upper Yukon stocks returning to lower and middle reaches of the region (53–61 km d^-1^). Somewhat slower movement rates were observed for fish returning to headwater areas (43–49 km d^-1^), which traveled in excess of 2,300 km upriver from Paimiut to reach their terminal tributaries.

**Table 3:** Travel distances associated with the last tracking station passed by radio-tagged Chinook salmon stocks returning to terminal tributaries in the Yukon River basin during 2002–2004.

			Distance from last station					
Region	Stock	N	Paimiut	Yukon^1^	Final^2^	Rate	SE	CV (%)
Tanana	Chena	56	1148	405	1–105	25.7	1.1	31.3
	Salcha	96	1147	404	0–168	23.5	0.6	23.8
	Goodpaster	52	1243	500	32–188	24.3	0.7	20.2
Yukon Flats	Chandalar	26	1205	25	6–222	52.2	2.2	21.6
	Sheenjek	10	1312	80	57–188	52.7	1.1	6.9
Up. Yukon	Klondike^3^	24	1702	(74)	38–114	60.5	1.4	11.2
	Stewart	56	1900	21	7–622	52.7	1.0	13.9
	Pelly	97	2063	15	44–641	58.5	0.8	13.4
	Little Salmon^3^	11	2135	(93)	22–57	41.3	1.9	15.2
	Big Salmon	56	2319	30	18–155	42.9	1.0	16.9
	Teslin^3^	81	2333	(6)	9–526	48.8	1.0	18.1

Distances (km) from the last station to 1) the first station in the lower basin (Paimiut), 2) the Yukon River main stem (Yukon), and 3) spawning sites within the tributary (Final) are presented. Stocks with the last station located on the Yukon River main stem are indicated, with the distance still to travel to reach the tributary in parentheses. The number of fish, movement rate (km d^-1^) for the reach (from the previous station to the last station), standard error (SE) and coefficient of variation (CV) are also presented. Stocks represented by less than 10 fish or lacking spawning sites information are not listed.

^1^Confluence of the Yukon River and the terminal tributary.

^2^Distance traveled from last station to spawning sites within the tributary (final location of fish). Reflects distance from the tributary mouth to spawning sites for stocks with last station on Yukon River main stem.

^3^Final station located on Yukon River main stem downriver from the terminal tributary.

#### Stock differences

Stocks returning to the same region generally exhibited similar migratory patterns. This was particularly true for Tanana and upper basin stocks (**Fig 3**). Fish returning to the Goodpaster River were only nominally faster than the other Tanana stocks. Movement rates for the two principal stocks in the Yukon Flats were essentially the same. Migratory patterns were comparable for the four principal Upper Yukon stocks (Stewart River, Pelly River, Big Salmon River, and Teslin River). These stocks comprised between 61–77% of the regional return based on stock composition estimates [15]. Stock-specific differences in the Upper Yukon were most apparent as the fish approached their terminal tributaries, particularly for stocks traveling farther upriver (**Fig 3**).

The migratory patterns of stocks returning to the Middle Yukon were less similar than those observed in other regions. Movement rates declined dramatically for Nulato River and Gisasa River fish as they approached their final destination, whereas Tozitna River fish exhibited a modest reduction in swimming speed (from 54 to 49 km d^-1^) during the later stages of the migration (**Fig 3**).

#### Within-stock differences

**Fig 4:**
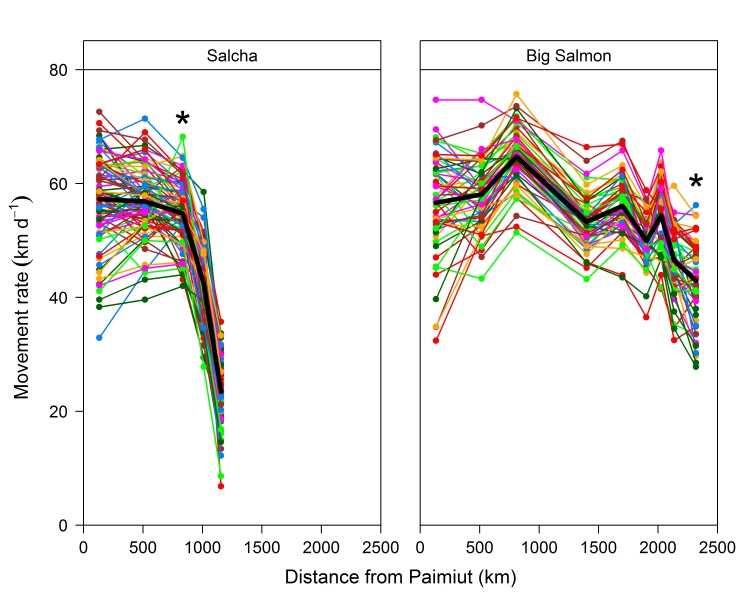
Movement rates for individual Yukon River Chinook salmon returning to the Salcha and Big Salmon rivers during 2002–2004. Black line indicates the average movement rate at each station. The first station passed after leaving the Yukon River main stem is also indicated (*).

In contrast to the similarities observed among stocks within the same region, there was considerable variation in the migratory patterns exhibited among individual fish within a stock. Individual movement rates at sequential stations varied widely as illustrated by fish returning to the Salcha (Tanana) and Big Salmon (Upper Yukon) rivers (**Fig 4**). Other stocks within the basin also exhibited considerable variation among fish (**S1 Fig**). However, the migratory patterns exhibited by the fish generally reflected the average migratory pattern exhibited by the stock. For example, most fish returning to the Salcha River exhibited consistent movement rates at the first three stations, followed by a pronounced decline at the fourth and fifth station (**Fig 4**). The tendency for individuals within a stock to exhibit the same general migratory pattern (*i*.*e*. increasing or decreasing movement rate) between sequential stations was particularly noticeable as the fish moved farther upriver from Paimiut.

**Fig 5:**
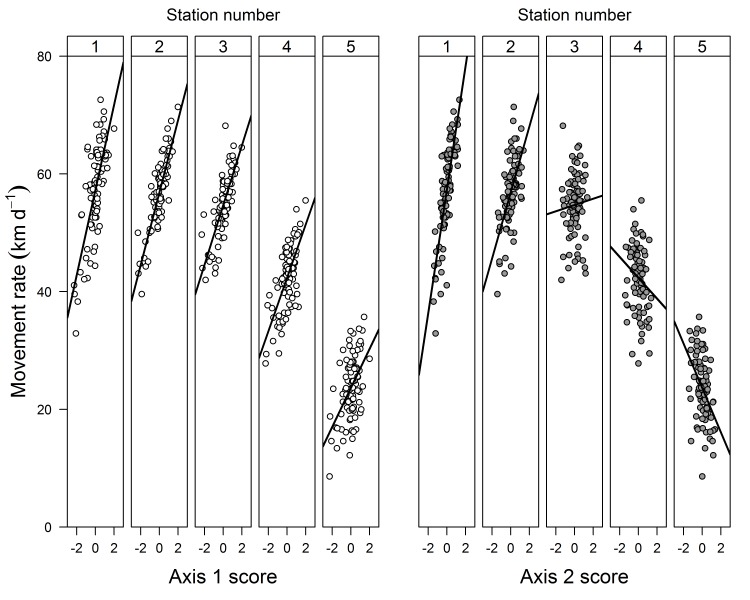
Relationship between movement rates and nonmetric multidimensional scaling (NMS) ordination scores for 96 Yukon River Chinook salmon returning to the Salcha River during 2002–2004. Both the Axis 1 (left panel) and Axis 2 (right panel) scores and associated regression lines are presented for the five sequential tracking stations located along the migratory route.

Two primary sources of variation in movement rate were identified for individual fish based on the within-stock ordinations of 11 stocks that met the sample criteria (**Table 4**). Usable ordinations were obtained for all the stocks examined as illustrated by Salcha River fish (**S3 Fig**). The dominant source of variation among individuals (represented by Axis 1) reflected the average movement rate of the fish, with the axis gradient ranging from slower fish (lower axis scores) to faster fish (higher axis scores). Simply stated, individual fish traveling slower in the lower basin exhibited consistently slower movement rates as they moved upriver compared to their faster moving counterparts, as reflected by the positive relationship between movement rate and the Axis 1 scores for the sequential stations (**Fig 5**). Similarly, fish with faster movement rates in the lower basin continued to display faster swimming speeds as they moved upriver relative to the slower fish. This source of variation among fish was often visually evident in the raw data (e.g., Big Salmon River fish in **Fig 4**). Axis 1 represented the dominant source of variation for all stocks analyzed, and on average explained 74% of the within-stock variation in movement rate associated with the multivariate data, ranging from 53% for White River fish to 94% for Chandalar River fish (**Table 4**).

**Table 4:** Yukon River Chinook salmon stocks analyzed using within-stock ordination to describe the migration patterns of individual fish returning to terminal tributaries based on average movement rates (km d^-1^) in sequential reaches of the basin.

						Pearson’s *r* ^*2*^		
Region	Stock	N	Rate	SE	CV (%)	Axis1	Axis2	Total
Tanana	Kantishna	20	54.9	1.2	9.7	0.919	0.079	0.998
	Chena	51	47.2	0.8	12.7	0.769	0.185	0.954
	Salcha	91	47.0	0.4	8.9	0.695	0.254	0.949
	Goodpaster	51	49.6	0.7	9.9	0.730	0.231	0.961
Yukon Flats	Chandalar	20	59.0	1.3	9.8	0.936	0.056	0.992
Upper Yukon	Klondike	24	63.1	0.9	6.8	0.739	0.177	0.916
	Stewart	48	58.7	0.8	9.2	0.726	0.250	0.976
	White	24	60.9	0.7	5.3	0.526	0.389	0.915
	Pelly	77	58.0	0.5	7.4	0.680	0.244	0.924
	Big Salmon	53	53.5	0.6	7.9	0.728	0.203	0.931
	Teslin	70	53.8	0.7	10.2	0.660	0.311	0.971

Pearson’s *r* ^2^ values represent the proportion of the multivariate data explained by the synthetic variables (axes).

**Fig 6:**
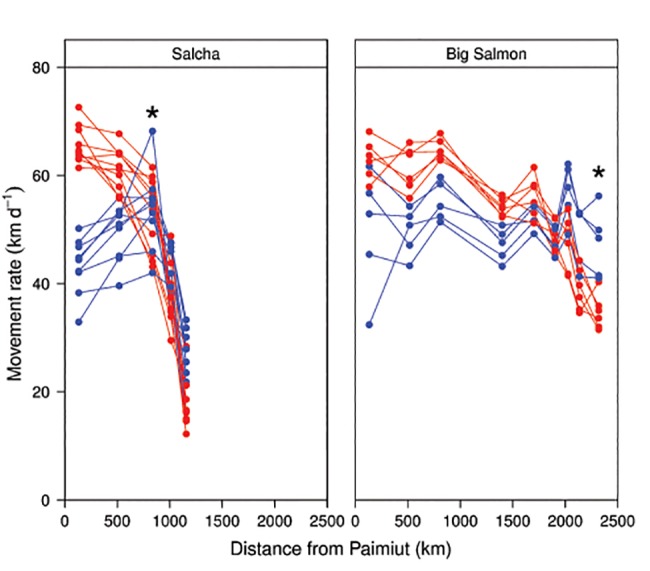
Movement rates for individual Yukon River Chinook salmon returning to the Salcha and Big Salmon rivers during 2002–2004. The fish shown include the upper 10% (dark circles, referred to as “hares”) and the lower 10% (open circles, referred to as “tortoises”) on the Axis 2 gradient from a nonmetric multidimensional scaling (NMS) ordination. The first station passed after leaving the Yukon River main stem is also indicated (*).

The second source of variation in movement rate (represented by Axis 2) reflected a shift in the relative swimming speeds of the individual fish as they progressed upriver. Although movement rates declined for nearly all of the fish within a stock during the migration, differences were observed in the pattern of the decline as the fish progressed upriver. Fish with faster movement rates in the lower river exhibited a pronounced decline in swimming speed as they moved upriver, as reflected by the progressive change from a positive relationship to a negative relationship between movement rate and the Axis 2 scores for the sequential stations (**Fig 5**). Conversely, fish moving slower in the lower river displayed a more gradual decline in movement rate. In many ways the two patterns were analogous to the proverbial characters in Aesop’s fable *The Hare and the Tortoise* [33], with some fish exhibiting hare-like movements (initially fast then slowing down) and others exhibiting more tortoise-like movements (slow and steady). The axis gradient ranged from fish exhibiting tortoise-like movements (lower axis scores) to those displaying hare-like movements (higher axis scores), hereafter designated as “tortoises” and “hares”. Based on within-stock comparisons of the outermost 10% of both the highest and lowest Axis 2 scores (shown in **S3 Fig** for the Salcha River), fish exhibiting the hare pattern ultimately displayed slower movement rates as they neared their terminal tributaries than fish with the tortoise pattern (**Fig 6**). Although direct comparisons are difficult due to differences among fish in run timing and other potentially confounding factors, the time taken by fish to reach their terminal tributaries was comparable for both tortoises and hares in spite of the substantially faster swimming speeds initially displayed by the hares. On average, Axis 2 explained 22% of the within-stock variation in movement rate represented by the multivariate data for all stocks analyzed, ranging from 6% for Chandalar River fish to 39% for White River fish (**Table 4**). Orthogonality was essentially 100% for Axis 2, suggesting that the information represented was not redundant in relation to Axis 1.

It is important to understand that the variation represented by Axis 1 and Axis 2 reflect a continuum in the migratory patterns exhibited by the fish, with every fish falling somewhere along both gradients. Simply stated, individual fish express different degrees of both the slow fish-fast fish and tortoise-hare pattern. Comparisons of the outermost 10% of both the highest and lowest Axis 1 scores were used to identify fish with movement rates that were either more consistently slower (negative values) or consistently faster (positive values) than other fish within the stock. Similarly, extreme Axis 2 scores represented fish exhibiting more tortoise-like or hare-like migratory patterns. For example, several Salcha River fish exhibiting the tortoise pattern (slow and steady) on Axis 2 also displayed the consistently slower pattern on Axis 1. Not surprisingly, fish exhibiting the hare pattern (initially fast, followed by a prominent decline) on Axis 2 displayed neither the consistently slower nor consistently faster pattern on Axis 1 (**S3 Fig**).

### Atypical Movements

Most of the fish tracked past multiple stations (2,560, 97.5%) exhibited continuous upriver movements and strong fidelity to the rivers they entered. Sixty-six (2.5%) fish deviated from this pattern (**Table 1**). Two types of anomalies were observed: fish that initially bypassed the terminal tributary that they ultimately selected and fish that entered more than one terminal tributary prior to spawning. Twenty-three fish (35% of the individuals exhibiting atypical movements) initially passed their final destination and continued upriver for varying distances, before reversing direction, swimming back downstream, and entering their terminal tributary. Some of these excursions were short, with fish moving less than 30 km upriver past their final destination before reversing direction and moving back downstream, whereas others were fairly extensive with fish traveling hundreds of kilometers out of their way (**Table 5**). The distances reported in **Table 5**represent minimum estimates, because it is not known how much farther upriver the fish ultimately traveled. The time spent upriver from the farthest station passed ranged from several hours to 8–9 days. Nine other fish displayed downriver movements, but were not tracked to terminal tributaries and were instead last located in mainstem areas or harvested in mainstem fisheries.

**Table 5:** Extra distance traveled (km) and time spent (d) by radio-tagged Chinook salmon that initially bypassed their terminal tributary and continued swimming upriver before reversing direction and traveling to their final destination in the Yukon River basin during 2002–2004.

Farthest (last) upriver station	Final destination	Fish^1^	Total fish	Extra distance traveled (km)^2^	Time upriver last station (d)
Yukon-Anvik	Anvik River	8 (11.3)	71	28	2.2 (0.1–8.0)
Yukon-Anvik	Bonasila River	1 (3.5)	29	70	6.2
Ruby	Nulato River	4 (9.8)	41	303	1.9 (0.1–4.7)
Ruby	Upper Koyukuk	1 (3.6)	28	110	6.7
Upper Tanana	Salcha River	1 (0.6)	164	110	0.1
Rapids	Tozitna River	3 (14.3)	21	179	4.0 (0.6–9.4)
Mid-Porcupine	Sheenjek River	1 (2.7)	37	303	5.0
Circle	Upper Porcupine	1 (2.2)	46	340	2.3
Circle	Chandalar River	1 (1.5)	65	368	1.2
Yukon-White	White River	2 (6.9)	29	13	0.5 (0.3–0.7)

The extra distance is a minimum estimate because the actual distance traveled by the fish past the farthest upriver station is unknown.

^1^Numbers of fish exhibiting bypass movements. Percentage of total number of fish with complete tracking records and tracked to final destination is in parentheses.

^2^Based on distance from terminal station (final destination) to farthest station upriver and return.

Thirty-four fish (52% of the individuals exhibiting atypical movements) were tracked to terminal tributaries, but subsequently left these rivers and traveled to other terminal tributaries within the basin (N = 31) or were harvested in upriver fisheries (N = 3). Most of these fish (23, 68%) were observed moving between two adjacent drainages (Bonasila River and Anvik River) in the Lower Yukon. The fish initially entered and remained in the lower reaches of the Bonasila River for a short time (usually for several hours, although one fish remained for several days) before moving back to the main stem and traveling to the Anvik River, located 17 km farther upriver. Similar incursions were observed in other tributaries, including the Nulato (N = 1) and Melozitna (N = 1) rivers in the Middle Yukon, Chena River (N = 1) in the Tanana, and the Stewart (N = 2) and Pelly (N = 1) rivers in the Upper Yukon, with fish entering and remaining in the lower reaches of the drainage for a limited period of time (typically < 1 day) before moving back to the main stem and resuming their upriver migration. These incidents were not limited to fish moving between nearby rivers. The fish that initially entered the Nulato River ultimately traveled to the Teslin River in the Upper Yukon, a distance of 1,950 km.

A small number of fish exhibited more convoluted movements, traveling substantial distances out of their way and initially entering rivers different from than their final destination. A fish tracked to the Sheenjek River in the Yukon Flats left this terminal tributary after 6 days and traveled downstream to the Chandalar River, a distance of over 100 km. Similarly, several fish that traveled to the upper reaches of the Porcupine River (a distance of over 337 km from the Yukon-Porcupine River confluence) ultimately returned to the main stem, traveled upriver to the upper reaches of the Upper Yukon, and were recovered in spawning areas in the Pelly and Teslin rivers.

### Fish Pulse Progression

**Fig 7:**
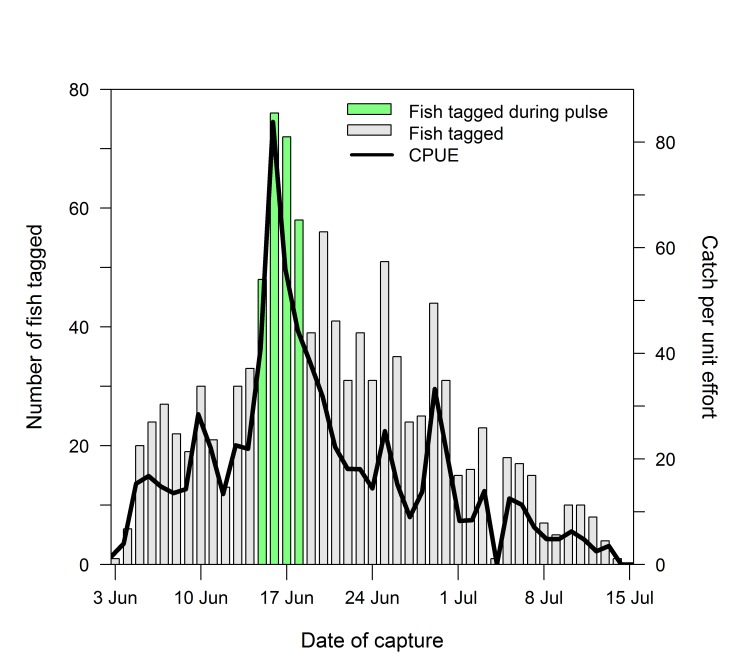
Daily number of Chinook salmon radio tagged in the lower Yukon River and daily catch per unit effort information for Chinook salmon captured at the Russian Mission tagging site during 2003. The fish tagged during the pulse associate with the peak of the run (15–18 June) are indicated.

**Fig 8:**
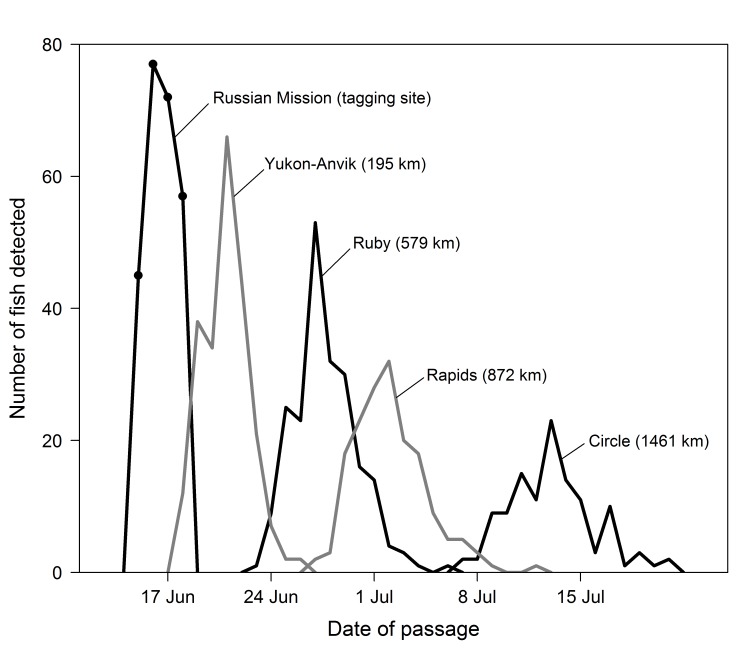
The upriver progression of fish tagged during the pulse of Chinook salmon moving past the Russian Mission tagging site during 15–18 June 2003 and located at successive upriver locations. The points indicate the daily number of fish tagged during the pulse. Distances upriver from the tagging site are in parentheses.

A total of 251 fish were radio tagged over a 4-day period (15–18 June) during the peak of the run in 2003 to provide information on the progression of this pulse of fish as it moved upriver from Russian Mission (**Fig 7**). Collectively, these fish passed the Yukon-Anvik River station over a 9-day period from 18–26 June. The time taken by the pulse to move past subsequent station sites continued to increase as the fish moved farther upriver from Russian Mission (**Fig 8**), with the fish taking 14–16 d to pass Ruby, Rapids, and Circle, and 19 d to pass the Yukon Border. Passage at mainstem stations farther upriver ranged from 16 to 24 days.

A similar pattern was observed for the portion of the pulse that left the main stem and continued up the Tanana River (N = 50). These fish passed the main river stations near Manley and Nenana over a 27-day and 24-day period, respectively (**Table 6**).

**Table 6:** Passage dates for Yukon River Chinook salmon radio tagged during the peak of the run and tracked upriver during 2003.

Location	Distance from tagging (km)	N	Passage dates	Passage duration (d)
Russian Mission (tagging site)	—	251	15–18 Jun	4
Yukon-Anvik River station	195	226	18–26 Jun	9
Ruby station	579	217	23 Jun—6 Jul	14
Manley station (Tanana River) ^1^	897	50	30 Jun—26 Jul	27
Nenana station (Tanana River)^1^	1,072	40	2–25 Jul	24
Rapids station (Yukon River)	872	168	27 Jun—12 Jul	16
Circle station (Yukon River)	1,461	116	7–21 Jul	15
Yukon Border station	1,764	104	11–29 Jul	19

Distances of the upriver locations from Russian Mission, number of fish, and numbers of days taken by the group of tagged fish to pass the sites are presented. The reduced sample size at the upriver locations reflects the harvest of radio-tagged fish in mainstem fisheries and the movement of fish into other terminal tributaries along the migratory route.

^1^Includes several fish tagged during the pulse, but moving substantially later past the site.

## Discussion

### Movement Rates

Chinook salmon returning to spawning areas in the Yukon River basin face a number of formidable challenges. In addition to the substantial distances traveled, the fish must respond to the physical and environmental constraints encountered along the way and reach spawning area when conditions are favorable. The fish must also arrive with sufficient energy reserves to avoid predation, select appropriate spawning sites, compete with other individuals, select mates, and successfully reproduce. The ability to accomplish these tasks is implicitly tied to the movements exhibited by the fish. Slower swimming speeds use less energy, but may be inadequate in relation to the time available to complete the migration [34]. Optimal swimming speed (*i*.*e*. minimum cost per distance traveled) for salmon has been estimated as one body length per second [35] or approximately 1.8 km/h^-1^ (43 km d^-1^) for an average sized Chinook salmon in the Yukon River. Swimming at optimal speed would be particularly advantageous for upper basin fish considering the extreme distances traveled, although environmental and temporal constraints likely compel the fish to swim in a less energy-efficient manner.

Most upper river fish displayed fast movement rates, ranging from 45 to 66 km d^-1^, suggesting that these fish were moving well above optimal swimming speed until the later stages of the migration. Movement rates for fish returning to spawning areas in the lower basin were substantially slower, ranging from 26 to 40 km d^-1^. The slower swimming speeds were likely related to the shorter distances these fish traveled and the reduced time needed to reach their final destination. The movement rates exhibited by lower basin fish were also more variable than those displayed by stocks returning to spawning areas farther upriver (**Table 2**). The compressed run timing of the return (~ 6 weeks [15]), extended distances traveled (in some cases over 3,000 km from the tagging area), and the need to reach terminal sites within a restricted period of time undoubtedly limits the swimming options available to fish traveling farther upriver, and may account for the reduced variability observed in upper basin stocks. Although precise timing information is not available on spawning within the basin, information from spawning ground surveys suggests that the peak of spawning in the different regions is generally confined to a 2–3 week period, with spawning in the upper basin occurring later in the season compared to lower and middle basin tributaries [15]. This narrow window (presumably related to environmental constraints associated with access to spawning grounds, spawning conditions within these areas, and offspring survival) may explain the compressed run timing and rapid movement rates exhibited by the return.

#### Comparisons with other rivers

Comparable information on the movement rates of Chinook salmon in other large river systems is limited due to the logistical challenges and costs associated with large-scale monitoring programs. Telemetry studies have been conducted on Chinook salmon in the Columbia River [13], [36], [37], [38] and provide useful comparisons. Both Yukon River and Columbia River studies encompassed large river basins, relied on tracking stations located on principal migratory routes and spawning tributaries, and tracked large numbers of radio-tagged fish to terminal spawning areas. Swimming speeds in free-flowing reaches of the Columbia River were substantially slower than observed during the present study, with most spring-summer Chinook salmon migrating at rates between 10 and 30 km d^-1^ [13]. Median movement rates in low gradient reaches of the Columbia River main stem (Hanford Reach, 553–639 km from the river mouth) were consistently < 40 km d^-1^, whereas rates in the lower Snake River (759 km from the river mouth) ranged between mid-30 and mid-40 km d^-1^. Median movement rates in other reaches of the basin were substantially less. Similar to our study, lower basin stocks generally displayed slower movement rates. Fall Chinook salmon exhibited a similar pattern, with median movement rates of approximately 38 km d^-1^ in the lower reaches of the basin [37]. Weekly movement rates were mostly between 30 and 45 km d^-1^ when water temperatures were below 21°C, but decreased by about 50% at higher temperatures [37].

Differences between the two river basins potentially explain some of the disparity observed in movement rates. Compared to the largely pristine and free-flowing conditions in the Yukon River basin, the Columbia River is heavily regulated, with controlled flows and inundated reaches associated with the numerous hydroelectric dams located throughout the drainage [39]. These structures have fundamentally altered the hydrological characteristics of the system, and resulted in diminished summer discharge, earlier warming of the lower river, higher peak temperatures, and later cooling in the fall [40], [41]. The consequences of these changes on migrating salmon vary widely between species and years, and are not well understood [38]. In contrast to the comparatively slow movement rates in riverine areas, Chinook salmon displayed rapid movements through impounded reaches, likely due the reduced water velocities encountered. Median movement rates in reservoirs associated with the lower Columbia River and Snake River dams ranged from 47–77 km d^-1^, and were influenced by run timing, distance traveled, river discharge, and water temperature [38].

In addition to other anthropogenic effects associated with the numerous cities and human activities along the Columbia River, the origin of the fish may also play a role. Chinook salmon returns to the Columbia River are composed of both wild and hatchery fish [42], whereas those returning to the Yukon River are almost exclusively from wild stocks. Differences in migratory movements have been reported between wild and hatchery fish [36], [43], [44] and may explain some of the disparity in movement rates observed between the Yukon River and Columbia River studies.

Chinook salmon returning to the Yukon River are also near the northern extent of their range, and may be environmentally constrained to a narrow migratory window, necessitating faster swimming speeds to reach terminal tributaries when spawning conditions are optimal. In contrast, Chinook salmon runs in the southern portion of their North American range extend throughout most of the year [42], [45], [46], [47], and likely face a less restricted range of environmental conditions and migratory options. Chinook salmon returning to the Copper River in south-central Alaska also exhibited slower movement rates, ranging from approximately 4 km d^-1^ in the lower river to 14 km d^-1^ in the upper reaches of the basin. However, this drainage is highly glacial and moderate in size, with the main river flowing < 300 km from its headwaters to the sea, and the heavy silt loads and shorter distances traveled by the fish are likely contributing factors. Movement rates for Chinook salmon returning to the relatively clear, moderately sized Klamath River (located within the southern portion of the range) averaged 21 km d^-1^ [48].

### Migratory Patterns

#### Regional and stock differences

Yukon River Chinook salmon displayed a variety of migratory patterns, with pronounced differences among the regional and stock-specific components of the return. Movement rates in lower reaches of the main stem were remarkably similar for upper river stocks, with pronounced differences observed as the fish continued upriver or left the main stem. Upper Koyukuk and Upper Porcupine fish swam considerably faster after leaving the main stem. River size, discharge, and turbidity were noticeably less in both of these drainages compared to the main stem, which undoubtedly provided less arduous swimming conditions for the fish. However, proximity to spawning areas was likely a contributing factor with faster swimming speeds observed for fish traveling farther upriver, as demonstrated by the differences in Koyukuk River fish returning to the lower (Gisasa River) and upper reaches of the drainage.

Conversely, a pronounced decline in movement rate was exhibited by Tanana stocks after leaving the main stem. These fish traveled 150–500 km farther upriver to reach their terminal tributary, suggesting that proximity to spawning areas was not the only contributing factor. Although discharge in the Tanana River was substantially less than in the main stem [15], the river is extremely turbid from glacial runoff which may have impacted swimming performance. The reduced movement rates may also reflect efforts to conserve energy reserves during the final stages of the migration or searching behavior as the fish attempt to detect environmental cues associated with their terminal tributary.

Fish that remained in the main stem for most of their upriver migration (e.g., Yukon Flats and Upper Yukon stocks) were characterized by a gradual but erratic decline in movement rate. Periodic increases in swimming speed were observed at varying distances from their final destination, suggesting that changes in the migratory pattern were more likely related to the geomorphic or hydrological characteristics of the river rather than the physiological condition of the fish or proximity to spawning areas. For example, faster movement rates were exhibited by the fish after passing the Yukon-Tanana River confluence; river discharge and turbidity upstream from this site were substantially less due to the lack of flow from the Tanana River, which may explain this pattern. Similarly, movement rates declined for over 99% of the fish traveling through the highly braided Yukon Flats. The subsequent increase displayed by fish after leaving this area suggests that the slower swimming speeds were in response to the physical features and hydrological conditions encountered.

Yukon Flats and Upper Yukon stocks did not exhibit a conspicuous decline in movement rate as they approached their terminal tributaries. For example, the movement rates of fish traveling through the Yukon Flats were comparable for both those stocks returning to this area to spawn and Upper Yukon fish with hundreds of kilometers still to travel. Slower movements by salmon approaching spawning areas are often attributed to increased efforts by the fish to search for and locate spawning areas. The lack of a prominent reduction in movement rate by upper basin stocks suggests that these fish were either not having difficulty recognizing the environmental cues used to home to their final destination (*i*.*e*. extensive searching was not necessary) or that these cues were not readily apparent until the fish were in the general vicinity due to the large size and substantial discharge associated with the main stem. Although movement rates for upper basin stocks generally declined as the fish progressed upriver, swimming speed for several headwater stocks actually increased during the final stage of their mainstem migration. In addition to the reduced river size and discharge in these reaches, which presumably afforded less challenging swimming conditions, proximity to spawning areas may be a contributing factor.

The migratory patterns of stocks returning to the same region were remarkably similar for Tanana, Yukon Flats, and Upper Yukon fish. These similarities suggest that the differences in movement rate observed in sequential reaches of the basin were not unique to certain stocks (*e*.*g*. reduced swimming speed in the Yukon Flats was displayed by all stocks passing through this area). Differences observed among Middle Yukon stocks were at least partly due to less extensive station coverage in the lower basin, and the spatial, geomorphic, and hydrological conditions encountered by the fish. A review of the literature did not reveal comparable information on the migratory patterns of Chinook salmon stocks in other river systems. For example, Keefer *et al*. [13] reported on the movement rates of spring-summer Chinook salmon aggregates in selected reaches of the Columbia River, but did not describe the sequential movements of these fish within the basin.

#### Within-stock differences

The migratory patterns exhibited by fish within a stock reflected two primary sources of variation, characterized by gradients ranging from slow fish to fast fish and from tortoise-like to hare-like behavior. The slow fish-fast fish gradient explained most of the variation among individual fish and was readily apparent when graphically comparing within-stock movements. Although the fish tended to reflect the general migratory pattern of the stock, the range in movement rate between the slowest and fastest individuals was often considerable. Substantial variation was also explained by the tortoise-hare gradient, ranging from 19–25% and 20–31% for the principal Tanana and Upper Yukon stocks, respectively, which comprise the largest components of the return. However, this source of variation was less apparent when comparing groups of individual fish and would have been difficult to identify based solely on visual inspection of the data.

A review of the literature did not reveal any other studies that used multivariate ordination to describe sources of variation in the movement patterns of individual animals traveling along a linear trajectory, such as migrating fish in riverine environments. It is noteworthy that nearly all (92–99%) of the individual variation represented by the original within-stock movement data was explained by the same two sources of variation (gradients) for each of the stocks examined. The influence of these gradients was consistent across all of the stocks analyzed, suggesting that the same principal sources of variation existed regardless of stock-specific differences and the migratory routes of the fish. Given that salmon returning to distant spawning areas are (generally) similar physically, typically stop feeding and exhibit a catabolic state after entering freshwater, and must complete the migration within a finite period of time, the sources of variation observed for Yukon River Chinook salmon are likely exhibited to some extent by other populations with extended migrations in large rivers. Chinook salmon traveling substantially shorter distances may or may not exhibit these sources of variation, since these fish presumably have fewer constraints during the freshwater migration and a wider range of options (e.g., greater latitude in the swimming speeds utilized) for successfully completing the journey. In this study, stocks that traveled shorter distances did not pass enough stations to provide meaningful ordinations, limiting the opportunity to evaluate the effect of distance on the sources of variation.

### Atypical Movements

Few fish exhibited atypical movements, and most of these consisted of short exploratory incursions by the fish into rivers downstream from their final destination. Fish moving from the Bonasila River to the Anvik River (located in the Lower Yukon) were the most common example. These two rivers were in close proximity to each other (< 20 km), drained adjacent watersheds, and likely had water with similar chemical characteristics. The use of olfactory cues by adult salmon to home to natal rivers is well established [49], and the incursion of fish into nearby tributaries with similar characteristics is understandable and consistent with reports of interim use of non-natal tributaries in other rivers (see below). No fish were observed moving from the Anvik River to the Bonasila River, suggesting that the fish were searching for their terminal tributary as they moved upriver.

Conversely, few exploratory movements were observed for fish returning to the Tanana River, even though several terminal tributaries within the drainage support large Chinook salmon returns and drain similar watersheds. Similarly, only a few upper basin fish exhibited between-tributary movements even though numerous rivers and streams within these regions support spawning populations. Distances between these rivers were typically greater than 30 km and proximity may be a factor, although a small number of fish exhibited fairly convoluted movements, traveling substantial distances out of their way and initially entering rivers that were considerably different than their final destination.

Small numbers of fish initially bypassed their final destination and continued traveling upriver before doubling back. These movements were likely inadvertent (*i*.*e*. the fish failing to detect the environment cues from their natal rivers during the initial passage), since the extreme distances traveled by the fish represent an energetic cost and extraneous movements would presumably have a negative impact. Although some bypass movements were short (< 30 km), most were fairly extensive ranging from 70 to over 360 km.

#### Comparisons with other rivers

The frequency of atypical movements by Chinook salmon in the Yukon River was substantially less than observed in other river systems. Keefer *et al*. [43] reported that over 14% of the radio-tagged fish in their study exhibited between-tributary movements, and that a substantial percentage (> 75%) of the lower basin fish initially bypassed their natal rivers. Based on these observations, it was suggested that direct point-to-point movements by salmon *en route* to natal stream may be less common than previously thought. Since limited information was available on the migratory patterns of Chinook salmon in other large river systems, fine-scale movements of sockeye salmon *O*. *nerka* in a small Alaskan river [10], which displayed localized movements between small, lake-shore tributaries, were used to support this view. However, information from our study suggests that most Chinook salmon in large, free-flowing rivers exhibit fairly direct upriver movements. The placement of stations during our study was not designed to document small-scale movements in localized areas, but bypass movements and movements between adjacent tributaries supporting large Chinook salmon stocks were rare. In addition to the impounded and highly regulated nature of the drainage, Chinook salmon returns to the Columbia River are composed of both wild and hatchery stocks [42], which may impact the behavior and migratory movements displayed by the fish. Keefer *et al*. [43] noted that atypical movements were more frequently displayed by Chinook salmon originating from hatcheries, particularly when considering Snake River stocks in the upper basin. Anomalous movements were also greater for later run fish, particularly when river temperatures exceeded 19°C.

Several similarities were observed between the atypical migratory patterns displayed by Yukon River and Columbia River Chinook salmon. Between-tributary movements were most common in lower reaches of the basin where several tributaries entered the main stem in close proximity to each other. Keefer *et al*. [43] speculated that this pattern was related to greater mainstem flows and more complex mixtures of olfactory cues in the lower Columbia River compared to areas farther upriver where spawning tributaries were more widely spaced and river discharge substantially less. Although not entirely analogous to the Yukon River basin (*i*.*e*. numerous tributaries in the Tanana and upper basin supported spawning populations of Chinook salmon in close proximity), river discharge in upper reaches of the basin was substantially less and derived from fewer sources of water than in the lower river, undoubtedly providing less ambiguous olfactory cues for the returning fish.

Keefer *et al*. [43] noted that bypass movements were more common for lower basin stocks, particularly when terminal tributaries were near dams, suggesting that homing behavior was less precise in impounded reaches and large migratory corridors compared to smaller, free-flowing rivers where less searching would be needed to locate natal streams. Based on observations that migrating salmon tended to migrate along shorelines and orient on plumes of water from upriver spawning tributaries [50], [51], they also speculated that bypass movements may be associated with fish traveling on the opposite side of the river from their terminal tributary and missing the olfactory cues, particularly in mainstem areas with substantial flows. Tributary size and outflow may also be a factor. During our study, most (70%) of the fish that showed bypass movements returned to smaller tributaries flowing into lower and middle reaches of the basin. The main stem is sizable within these areas, and it is possible that the tributary discharge (and the associated olfactory signal used by the homing salmon) may periodically be obscured by the mainstem flow. In contrast, there is strong evidence that Tanana River fish start exhibiting bank orientation several hundred kilometers downstream of the Yukon-Tanana confluence [52] due to the substantial discharge and distinct river characteristics of this drainages, which may explain the lack of bypass movements by Tanana stocks within this section of the main stem.

### Assumptions and Data Interpretation

Movement data can be confounded by a number of factors, ranging from the collection methods used to assumptions related to the behavior of the fish. Recognizing these potential biases is fundamental to analyzing and interpreting the information. Movement rate was considered the most appropriate measure of upriver movement since it normalized differences in the distance traveled between stations, which in the case of this study ranged from 20 to 640 km. Due to the size of the basin and scope of the study, it was not possible to determine the actual pathway selected by the fish during the upriver migration. Therefore, the distance used to calculate movement rate was based on the assumption that fish were primarily traveling along the thalweg. This approach avoided underestimating distance by not transcribing migratory routes through areas inaccessible to fish (*e*.*g*. islands, dewatered channels), and avoided overestimating distance traveled by assuming that fish were not following the most circuitous route. This method likely provided accurate distance estimates in reaches where the river consisted primarily of the main channel with occasional side channels and sloughs (*i*.*e*. limited options for the migrating fish)—conditions typical for most of the basin—but was potentially less accurate in highly braided reaches where the fish had an opportunity to select a variety of pathways. “Slower” movement rates in braided areas could reflect the additional time needed by fish to move through the reach via a more circuitous route rather than a reduction in swimming speed.

Migrating salmon reportedly seek out and use current as a directional cue [53]. Remaining in the thalweg would presumably help the fish avoid circuitous or unsuccessful migratory routes, but the energetic costs of swimming against strong current can be substantial [54], [55]. However, free-flowing rivers are extremely dynamic and their irregular shapes and non-laminar flow often create a highly variable velocity gradient [56], which the fish may exploit to select pathways with less flow. Fine-scale movements were not routinely examined during this study due to the vast size of the basin, limited resources, and tracking efforts based primarily on widely spaced stations and periodic aerial surveys, which minimized the information collected in localized areas. However, limited boat tracking in the lower river suggests that the fish regularly shift from the main channel into adjoining eddies. Local fishers often fish main river eddies, further suggesting that these areas are frequently used by migrating salmon. The routine use of eddies while traveling along the thalweg would presumably reduce the energy expended by the fish while allowing them to stay in close proximity to the main channel.

It is not known whether the fish spend extended periods of time resting during the upriver migration. The movement rates estimated during this study were based on the assumption that the fish were continually moving upriver. Prolonged holding by the fish would bias these estimates low, and suggest that the fish were actually swimming at faster speeds. However, this seems unlikely given the rapid movement rates observed during this study, suggesting that holding by the fish during the upriver migration was limited. Delayed upriver movements have been reported in other river systems for salmon utilizing thermal refuges [37] and encountering velocity barriers. Rand and Hinch [57] found that Fraser River sockeye salmon passing a velocity barrier exhibited behavioral strategies that included periods of stasis punctuated by swimming bursts. However, Standen *et al*. [58] reported that pink salmon *O*. *gorbuscha* moving through reaches of the Fraser River with fast, turbulent flows generally displayed faster, less energy-efficient swimming patterns in spite of the increased energetic costs, presumably to minimize travel time through these areas since significant delays could affect arrival times on spawning grounds and negatively impact spawning success.

The location of certain stations within the basin periodically hampered regional and stock-specific comparisons. The geomorphology of the river is complex, and it was not always possible to select sites in the most ideal location (e.g., river confluences, transitional sites between river types). For example, no suitable station site was available near the Yukon-Porcupine River confluence due to the braided nature of the Yukon Flats. Instead, the initial Porcupine River station was located over 210 km upriver from the confluence and reflected movements in both the Yukon and Porcupine rivers. The number of stations deployed was also limited due to the equipment, installation, and operational costs, which restricted coverage in such a vast and remote river basin. This limitation was particularly evident for minor stocks. No stations were located in the upper reaches of the Koyukuk River, making it difficult to characterize the movements of the fish as they approached their final destination, although ancillary information from local fisheries and assessment projects occasionally made it possible to speculate on the migratory patterns. In spite of these issues, the system of stations used during the study was sufficient to determine the principal migratory patterns of the return.

### Applications to Management

Detailed information on salmon movements is often not available to fishery managers, and the methods for incorporating it into the decision making process not always straight forward. However, understanding the underlying patterns is fundamental to effectively managing migratory populations. When combined with information on run timing and stock structure, movement data can provide valuable insight into the status of the returns and the passage of fish through in-river fisheries. Movement data can be particularly important in large drainages with widely scattered fisheries where management actions in the lower river potentially impact harvests and escapement farther upstream.

The findings from this study illustrate the need to consider individual variation when managing migratory populations. The migratory patterns displayed by the fish were not monotonic. Instead, substantial individual variation in movement rate was observed, both in terms of magnitude and the temporal/spatial patterns exhibited by the fish, which may complicate efforts to model swimming behavior and manage in-river fisheries. The considerable individual variation observed also suggests that information based on small numbers of fish may not adequately reflect the range of migratory patterns exhibited by the larger population, and cautious should be taken not to over-interpret or generalize the patterns observed.

**Fig 9:**
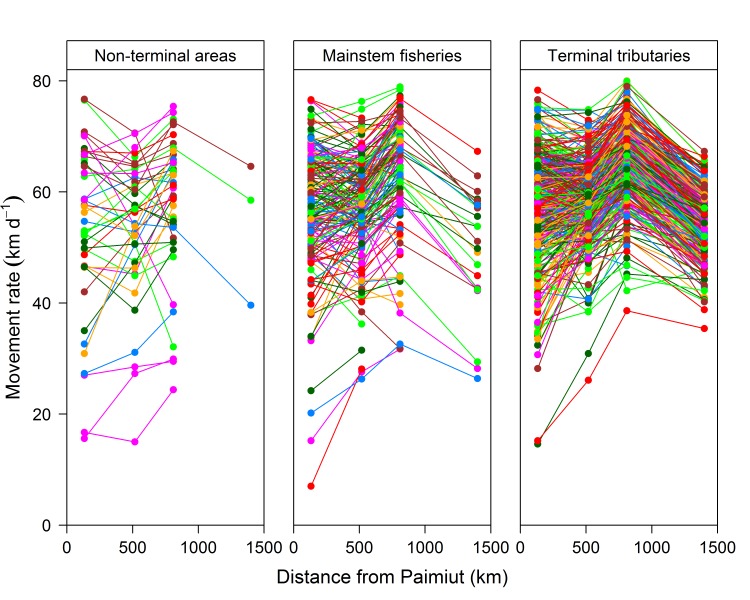
Movement rates for individual Yukon River Chinook salmon tracked to non-terminal areas (left panel), harvested in mainstem fisheries in the Yukon Flats (middle panel), or tracked to terminal spawning tributaries in the upper basin during 2002–2004.

Movement information can also be used to assess other management issues, including the impact of human activities, hydrological conditions, and the physical features within the basin, as well as biological constraints on the fish. For example, since the late 1990s there has been concern within the Yukon River basin over the presence of the fish parasite *Ichthyophonus* and its impact on Chinook salmon returns. Studies have suggested that infected fish succumb to the parasite while in-transit to spawning areas in the upper basin [59]. During our study, a number of fish were tracked to non-terminal reaches within the main stem [15]. The status of these fish is uncertain due to difficulties associated with accessing and sampling the sites. Although these individuals may have spawned in mainstem reaches (or nearby areas), they may also represent fish that died while in-transit to spawning areas farther upriver. Although disease and latent handling effects cannot be definitively ruled out, the migratory patterns of the fish tracked to non-terminal areas suggest that other factors were involved. Reduced stamina and progressively slower swimming speeds would be expected for fish in a weakened state or in the process of dying while in-transit to spawning areas farther upriver [60], [61]. However, most non-terminal fish did not exhibit this trend, exhibiting movement rates that were similar to those displayed by fish harvested in main stem fisheries or tracked to terminal tributaries in the upper basin (**Fig 9**).

The migratory patterns documented during the present study also provide a baseline for future comparisons. Reductions in fish size [62] and shifts in age composition to younger fish (based on data from [63]) have been reported within the basin and may affect the migratory patterns exhibited by the returns. Climate change is also an increasing concern, particularly in the Arctic due to polar amplification [64]. Salmon returns could be seriously affected by prolonged exposure to elevated water temperatures due to the extended spawning migrations in many northern rivers. Reported effects from increased temperatures on migrating salmon range from reduced stamina [65], pronounced shifts in run timing, swimming speeds, and increased use of thermal refuges [13], [37], [41], [66], and elevated mortality [67], [68]. The accelerated progression of disease and its increased severity under warmer temperature regimes [69] would also likely impact the swimming performance and stamina of the fish [60].

#### Fish pulses

The migratory similarities exhibited by stocks within the same region would seemingly provide a mechanism for tracking in-season movements and managing in-river fisheries. However, the underlying complexities exhibited by individual fish introduce a number of complications, as illustrated by the fish pulse monitored in 2003. Large pulses of Chinook salmon moving upriver through the main stem are routinely used by fishery managers to identify and target different components of the Yukon River return (D. Bergstrom, Alaska Department of Fish and Game, Anchorage, Alaska, personal communication). During years of low abundance, fisheries are closed as the pulses pass upriver through successive fisheries to reduce harvests and enhance spawning ground escapements. In addition to monitoring the relative magnitude of the run, GSI information is also collected for fish passing through the lower river [70] to provide in-season estimates of the regional composition of the return and to provide a means to minimize harvests on stocks of concern. However, the lack of synchronous movements within the pulse as the fish progress upriver can potentially impact the effectiveness of these types of management actions.

Large numbers of fish were tagged during the pronounced pulse in 2003 because it provided a strong signal that could be tracked upriver. The timing of the tagged fish as they passed successive sites along the migratory route suggests that pulses of fish are not exhibiting synchronous movements, and that subsequent signals detected farther upriver likely represent different combinations of fish. The temporal spread of the tagged fish would be expected to remain constant if the pulse was cohesive. Instead, the time taken by the fish to pass successive sites became progressively more protracted as the distance upriver increased.

This lack of temporal synchrony is not surprising considering the substantial variation exhibited among individual fish. Most of this variation was explained by the slow fish-fast fish gradient. This source of variation would tend to amplify spatial differences, with consistently faster fish tending to outdistance their slower moving counterparts over time. A strong pulse was still apparent at the upriver sites, but the composition of this aggregate likely consisted of a fraction of the original fish, as well as a combination of slower moving fish overtaken by the pulse and faster moving fish that had “joined” the pulse for a period of time. Although the variation explained by the tortoise-hare gradient was considerably less, this added complexity would further confound efforts to predict arrival times at locations farther upriver along the migratory route.

## Conclusions

Chinook salmon returns to the Yukon River displayed a variety of migratory patterns. Although most fish exhibited continuous upriver movements and strong fidelity to the terminal tributaries they entered, pronounced differences were observed among the regional components of the return, stocks, and individual fish. Fish traveling to terminal tributaries in the lower basin were uniformly slower than those traveling farther upstream, whereas upper river fish exhibited three distinct migratory patterns. Stocks returning to the same region generally exhibited a similar migratory pattern; however, the movements displayed by the individual fish were not monotonic. Most of this variation was explained by differences between consistently slower and faster moving fish, which would tend to amplify spatial and temporal differences—as was demonstrated by the lack of synchrony for the fish tagged within a prominent pulse of fish. Although the variation explained by relative changes in swimming speed (tortoise-hare gradient) was considerably less than the slow fish-fast fish gradient, this added complexity would further confound efforts to predict arrival times at locations farther upriver. Similarly, small numbers of fish exhibited short exploratory excursions before entering their terminal tributaries. Some of these movements were more convoluted with fish traveling hundreds of kilometers out of their way. The movement rates observed during this study were substantially faster and the percentage of fish exhibiting atypical movements considerably less than reported in more southerly drainages, and may reflect the pristine conditions within the basin, wild origins of the fish, and discrete run timing of the returns. Movement data can provide numerous insights into the status and management of salmon returns, particularly in large river drainages with widely scattered fisheries where management actions in the lower reaches can potentially impact salmon harvests and escapement farther upstream. However, the findings of this study illustrate the need to consider regional, stock, and individual differences when managing migratory populations, which may complicate efforts to model swimming behavior and manage in-river fisheries.

## Supporting Information

S1 Fig Movement rates for Chinook salmon returning to terminal tributaries in the Yukon River basin during 2002–2004. Black lines indicate the average movement rate at each station.
(EPS)

S2 Fig Movement rates for Chinook salmon harvested in fisheries or last located in non-terminal reaches of the Yukon River basin during 2002–2004. Black lines indicate the average movement rate at each station.
(EPS)

S3 Fig Nonmetric multidimensional scaling (NMS) ordination based on upriver movement rates of 96 Yukon River Chinook salmon returning to the Salcha River and recorded by five sequential tracking stations located along the migratory route during 2002–2004. The upper 10% (dark circles), central 80% (open circles), the lower 10% (dark triangles) of the Axis 2 scores of the fish are indicated.
(EPS)
