# Correction: Migratory Patterns of Wild Chinook Salmon *Oncorhynchus tshawytscha* Returning to a Large, Free-Flowing River Basin

**DOI:** 10.1371/journal.pone.0134191

**Published:** 2015-07-28

**Authors:** John H. Eiler, Allison N. Evans, Carl B. Schreck

There are multiple errors in the caption for Fig 6, “Movement rates for individual Yukon River Chinook salmon returning to the Salcha and Big Salmon rivers during 2002–2004.” Please see the complete, correct Fig 6 caption here.

**Fig 6:**
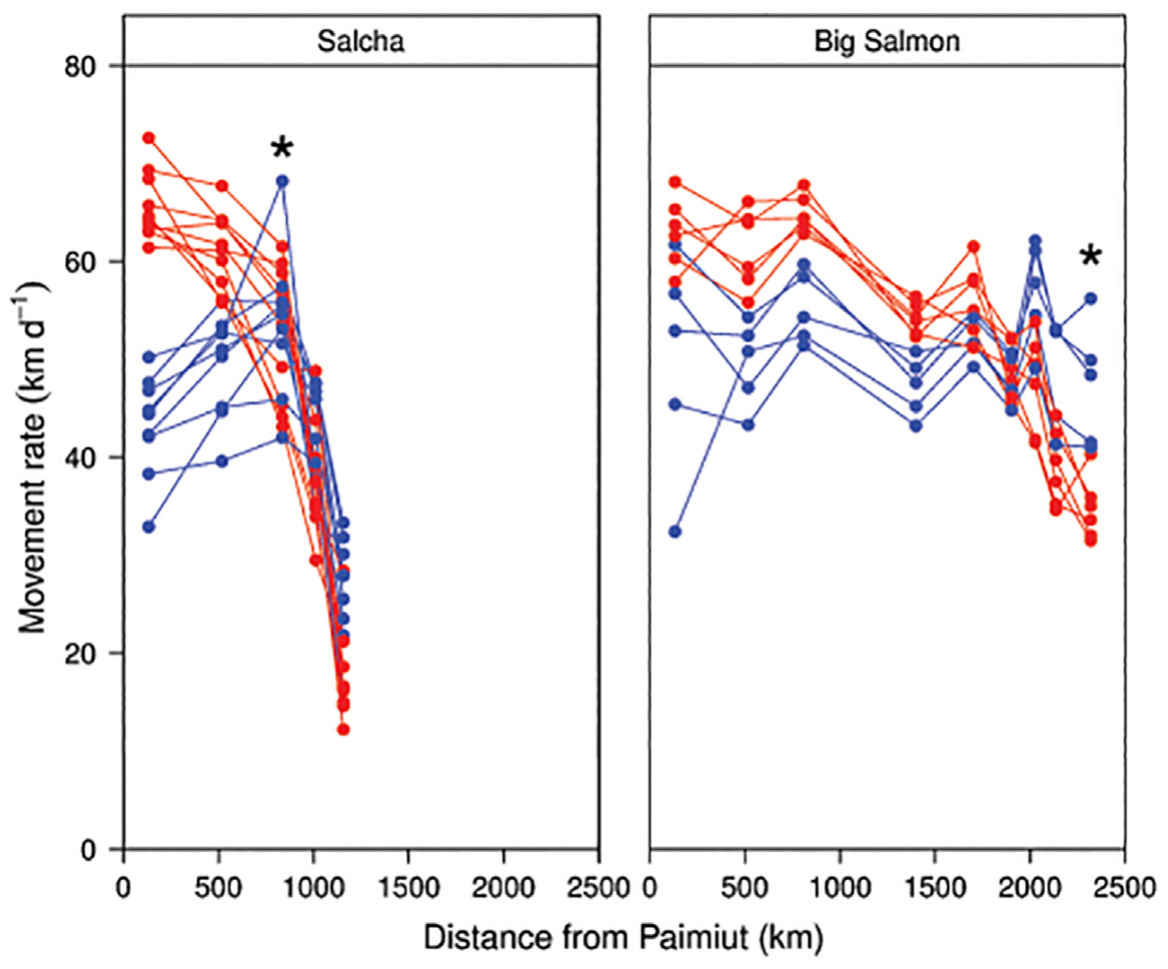
Movement rates for individual Yukon River Chinook salmon returning to the Salcha and Big Salmon rivers during 2002–2004. The fish shown include the upper 10% (red circles, referred to as “hares”) and the lower 10% (blue circles, referred to as “tortoises”) on the Axis 2 gradient from a nonmetric multidimensional scaling (NMS) ordination. The first station passed after leaving the Yukon River main stem is also indicated (*).
